# Immunomodulatory Effect of Tremella Polysaccharides against Cyclophosphamide-Induced Immunosuppression in Mice

**DOI:** 10.3390/molecules23020239

**Published:** 2018-01-25

**Authors:** Yalin Zhou, Xiaoyong Chen, Ruokun Yi, Guijie Li, Peng Sun, Yu Qian, Xin Zhao

**Affiliations:** 1 Department of Biological and Chemical Engineering, Chongqing University of Education, Chongqing 400067, China; chenxiaoyong522@163.com (X.C.); yirk@cque.edu.cn (R.Y.); quajetlee@gmail.com (G.L.); lazhuyu2@163.com (P.S.); qianyubaby@126.com (Y.Q.); 2 Chongqing Collaborative Innovation Center for Functional Food, Chongqing University of Education, Chongqing 400067, China; 3 Chongqing Engineering Research Center of Functional Food, Chongqing University of Education, Chongqing 400067, China; 4 Chongqing Engineering Laboratory for Research and Development of Functional Food, Chongqing University of Education, Chongqing 400067, China

**Keywords:** tremella polysaccharides, immunological activity, cyclophosphamide

## Abstract

Polysaccharides are closely associated with immune regulation, but there are different polysaccharide effects from different sources. In this study, the aim was to investigate the effect of tremella polysaccharides (TP) in cyclophosphamide-induced immunodeficient mice. We observed the thymus and spleen index, liver and spleen pathological changes, and the levels of IL-2, IL-12, INF-γ, TGF-β and Ig G in serum, and we also noted the mRNA expression of IL-1β, IL-4, IL-12 and TGF-β in liver and spleen. Besides, we also measured the best effects of different doses of TP (Low-TP was 20 mg/kg·BW, Middle-TP was 40 mg/kg·BW, and High-TP was 80 mg/kg·BW) on cyclophosphamide-induced immunosuppressed mice. The results were remarkable, and suggested that TP had a significant effect for enhancing immunity in cyclophosphamide-induced immunosuppression, and the immune enhancement of High-TP had the best results in TP-treated mice. It could significantly increase the thymus and spleen index, alleviate pathological features of immunosuppression such as the arrangement of liver sinusoid and hepatic plates was disordered, massive inflammatory cells infiltrated and fatty degeneration of hepatocytes in liver, and red pulp and white pulp were intermixed, splenic corpuscles demolished and disappeared, splenic sinusoid extended, and lymphocytes of spleen were reduced in spleen. Besides, it could also up-regulate serum levels of IL-2, IL-12, INF-γ and Ig G, reduce the level of TGF-β in serum, markedly promote mRNA expression of IL-1β, IL-4 and IL-12 in liver and spleen, and suppress mRNA expression of TGF-β. Above all, TP showed preventive effect for cyclophosphamide-induced immunosuppressed mice.

## 1. Introduction

Polysaccharides are natural macromolecular compounds that exist widely in various animals, plants, microorganisms, lichens and seaweed. In addition, Polysaccharides have many different kinds of biological activities, including regulating immune function, anti-tumor, anti-oxidant and anti-inflammatory activities [1,2,3,4,5]. Among its many biological activities, the immune activity of polysaccharides is recognized as the remarkable feature and it can modulate the body’s immune functions by regulating immune organs, immune cells and immune molecules [6]. Previously, water-soluble yam polysaccharides could elevate the spleen and thymus index [7]. Polysaccharides from ginseng leaves could significantly promote the activation of macrophages and NK cells [5]. Polysaccharides from cyrtomium macrophyllum (CMP) had a good immune regulative effect by improving NO production, TNF-α secretion and iNOS protein of RAW264.7 cells [3]. In addition, polysaccharides from Chinese rice wine could also significantly increase IL-6, INF-γ, TNF-α, Ig A, Ig M, Ig G and complement C3, complement C4 in mice serum [8].

Besides, Polysaccharides have been considered as important immunostimulant agents because of their immune activity with no significant side effects. Tremella is a well-known edible fungus with pharmacological action in oriental countries [9]. The polysaccharides from tremella possess extensive bioactivities and functions, including anti-cancer, tumor inhibition and immune regulation [10,11,12]. However, the polysaccharides from tremella in different regions were different so the bioactivities and functions of polysaccharides also had differences. Tremella Polysaccharides derive from Tremella fruitbodies and silver cell spore fermentation. The main chain is mannan, which is composed of a α-(1-3)-glycosidic bond. The main chain has 2, 4 and 6 positions which are connected with glucose, xylose, fucose and common uronic acid residues. Its active center is the common structure part of alphaα-(1-3)-mannan [13]. Therefore, combined with other authors’ results, further research on tremella polysaccharides from new regions (the tremella polysaccharides have not been studied) will be a useful way to find tremella polysaccharides with better bioactivities and functions.

Cyclophosphamide (CY), an alkylating agent, has been most widely used to treat autoimmune diseases and cancer [14,15]. CY is also a cytotoxic drug, but it has no activity in vitro, and it can damage the structure of DNA, cut off its copy that can lead to cell death in vivo [16,17]. Research showed that the long-term use of CY had harmful side effects, such as immunosuppression, myelosuppression and leucopenia [18]. Most importantly, immunosuppression was one of the major side effects of using high doses of CY. Therefore, CY was often used to construct murine models of immunosuppression. For example, based on the murine model of immunosuppression by CY, previous research had revealed that the effect of millimeter waves on cyclophosphamide-induced suppression of the Immune System [19], *Lactobacillus plantarum* NCU116 could remit cyclophosphamide-induced immunosuppression [20], and the glycopeptides from *Paecilomyces sinensis* had a good immunostimulatory activity [21].

In this study, the aim was to evaluate the role of tremella polysaccharides (TP) on improving immune function using a cyclophosphamide-induced immunosuppressed murine model. In addition, immune organ index, pathological features of immune organs, immune-related cytokines in serum, as well as the mRNA expression levels of immune-related genes in spleen and liver were also examined to evaluate the beneficial effects of TP. Tremella polysaccharide may have a good immune regulation effect. By detecting this effect, it will be more beneficial to the development and utilization of tremella polysaccharide.

## 2. Materials and Methods

### 2.1. Materials and Chemicals

Tremella polysaccharide was purchased from Xi’an Tongjiang Biological Science and Technology Co., Ltd. (with a purity of ≥98%, Xi’an, Shanxi, China), the polysaccharide was derived from Tremella which was produced in Sichuan, China [22]. Cyclophosphamide was purchased from Shanghai Ryon Biological Technology Co., Ltd (Shanghai, China). ELISA kits for immunoglobulin G (IgG), interleukin-2 (IL-2), interleukin-12 (IL-12), transform growth factor-β (TGF-β) and interferon-γ (INF-γ) were obtained from Shanghai Enzyme-linked Biotechnology Co., Ltd (Shanghai, China). TRIzol^®^ Reagent, RevertAid First Stand cDNA Synthesis Kit and SYBR^®^ Select Master Mix were provided by Thermo Fisher Scientific (New York, NY, USA).

### 2.2. Animal Experiments

50 Kunming mice (25 males and 25 females, six weeks old) were purchased from the Experimental Animal Center of Chongqing Medical University (Chongqing, China). All mice were fed with a standard mice chow diet and water ad libitum in special conditions (temperature 25 ± 2 °C, relative humidity 50 ± 5%, and 12 h light-dark cycle). After a week, fifty mice were divided into five groups according to body weight (each group had 5 male mice and 5 female mice), Normal group (Normal), Model group (Model), Low concentration group of TP (Low-TP), Middle concentration group of TP (Middle-TP) and High concentration group of TP (High-TP) respectively. Three days before the experiment, cyclophosphamide (200 mg/kg·BW) was administered to all mice by intraperitoneal injection (i.p.) besides Normal group.

**Figure 1:**
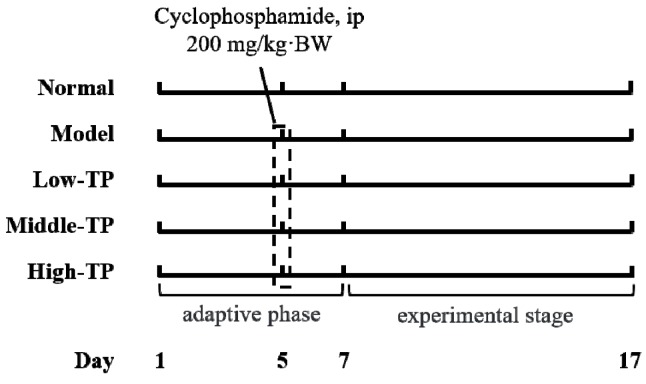
Experimental process in this study.

After the experiment commenced, the Normal and Model were given the same dose of physiological saline water by i.p. every day, then the Low-TP (20 mg/kg·BW), Middle-TP (40 mg/kg·BW) and High-TP (80 mg/kg·BW) were administered with the corresponding concentration TP by i.p., and the work lasted 10 days (Figure 1). 12 h after the last i.p., we measured body weight of each mouse, and then collected blood from the retro-orbital axis while under anesthesia and immediately took the liver, thymus and spleen after that mice were killed and weighted thymus and spleen to calculate immune organ index [23] (thymus index = thymus weight (mg) × 10/body weight (g), spleen index = spleen weight (mg) × 10/body weight (g)). Blood serum was collected with 3000 r/min for 15 min at 4 °C. A small amount of liver and spleen samples were used to tissue biopsies, and the surplus liver and spleen samples were transferred to ultra-low temperature freezer after liquid nitrogen flash freezer for standby application. The protocol for these experiments was approved by the Animal Ethics Committee of Chongqing Medical University and the animal permit number is SYXK (Yu) 2017-0001.

### 2.3. Histological Observations

The liver and spleen samples were fixed in 10% (*v*/*v*) buffered formaldehyde, and embedded with paraffin wax in molds. 4 µm slices were cut from paraffin blocks, then the slices were stained with hematoxylin and eosin (HE) and observed by using a microscope (Olympus BX43, Olympus Co., Tokyo, Japan).

### 2.4. Measurement of Biochemical Parameters in the Serum

The levels of IgG, IL-2, IL-12, TGF-β and INF-γ in the serum were measured by enzyme-linked immunosorbent assay kits (Shanghai Enzyme-linked Biotechnology Co., Ltd., Shanghai, China).

### 2.5. qRT-PCR Analysis

Total RNA was extracted from liver and spleen with TRIzol^®^ Reagent, and the concentration and purity of RNA were determined using a micro-spectrophotometer (Nano-300, Hangzhou Allsheng Instruments CO., Ltd., Hangzhou, Zhejiang, China). 1 µg total RNA was reverse transcribed to cDNA using RevertAid First Stand cDNA Synthesis Kit, and the concrete steps were performed in strict accordance with product manufacturer’s instructions. The mRNA expression levels of interleukin-1β (IL-1β), interleukin-4 (IL-4), interleukin-12 (IL-12) and transforming growth factor-β (TGF-β) were quantified by qRT-PCR (quantitative real-time polymerase chain reaction) with SYBR^®^ Select Master Mix and Step One Plus Real-Time PCR System (Thermo Fisher Scientific, New York, NY, USA). The qRT-PCR Systems were as follow: cDNA templates 1 µL, SYBR^®^ Select Master Mix 10 µL, each of the primer (10 µM) 1 µL, ddH2O 7 µL. Thermal cycling conditions were as follows: an initial incubation at 95 °C for 10 min to activate the polymerase followed by 40 cycles of 95 °C for 15 s, 60 °C for 1 min. The melting curve analysis was included after 40 cycles to verify the primer specificity by heating from 60 °C to 95 °C with fluorescence measured. The primers used for qRT-PCR are listed in Table 1. We used the housekeeping gene *β-actin* as the internal standard.

### 2.6. Statistical Analysis

The experimental dates analysis was performed with Excel 2010 (Redmond, WA, USA) and Graphpad Prism 7.0 (GraphPad Software Inc., La Jolla, CA, USA), and which were expressed as mean ± standard deviation. The differences among the groups were evaluated by ANOVA with the Dunnett’s test for post hoc analysis.

## 3. Results

### 3.1. Immune Organ Index

**Figure 2:**
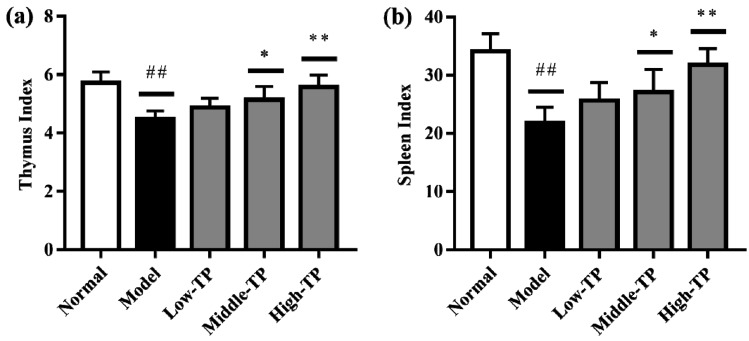
Effect of TP on immune organ indexes, (**a**) thymus index and (**b**) spleen index. ## *p* < 0.01, compared to Normal. * *p* < 0.05; ** *p* < 0.01, compared to Model.

As shown in Figure 2, compared to Normal, Model had significantly lower the thymus (Figure 2a) and spleen index (Figure 2b) (## *p* < 0.01). In addition, compared to the Model, the thymus and spleen index of Low-TP had no significant change, but it significantly increased in Middle-TP and High-TP (Figure 2a,b; * *p* < 0.05; ** *p* < 0.01). The results suggested that cyclophosphamide can sharply reduce the thymus and spleen index, and high dose TP can effectively protect the thymus and spleen index decreased significantly.

### 3.2. Histological Observations of Liver and Spleen

**Figure 3:**
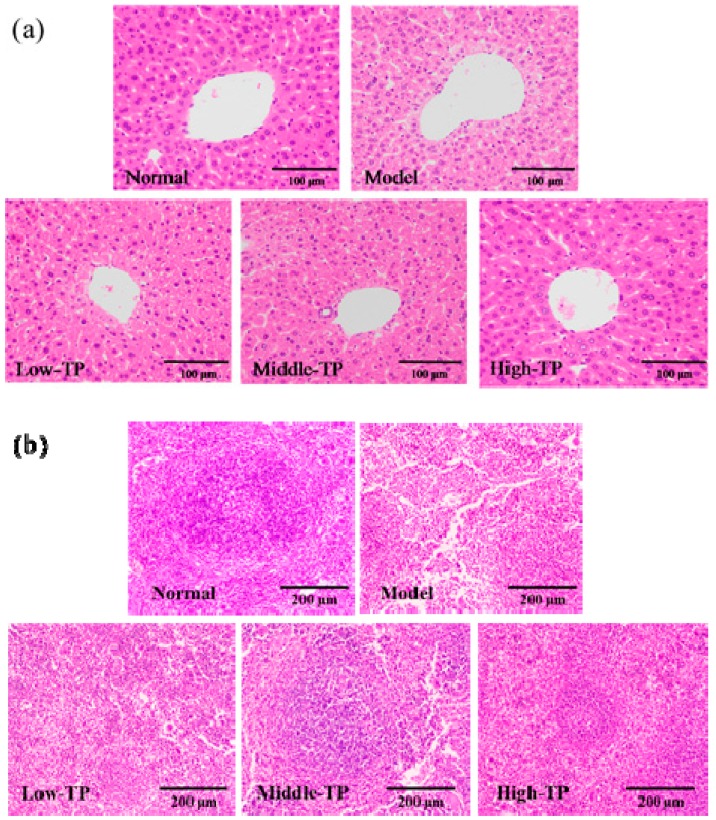
Histopathology observation of liver (**a**) 200× magnification) and Spleen (**b**) 100× magnification.

To further evaluate the effect of TP, the histopathology of the liver and spleen was evaluated with H&E staining. As shown in Figure 3a, the photomicrographs of liver in the model indicated that the arrangement of liver sinusoid and hepatic plates was disordered, massive inflammatory cells infiltrated and fatty degeneration of hepatocytes. After TP treated, the disordered arrangement of liver sinusoid and hepatic plates, inflammatory cells infiltrated and fatty degeneration of hepatocytes was relieved in different degrees compared with Model, and the photomicrographs of liver in High-TP was similar to that in Normal. As shown in Figure 3b, the photomicrographs of spleen in Model show that red pulp and white pulp are intermixed, splenic corpuscles demolished and disappeared, splenic sinusoid extended, and lymphocytes of spleen were reduced. After treated with TP, we could see these features in Low-TP and Middle-TP easily, but they improved in High-TP. It was remarkable that the photomicrographs of spleen in High-TP only showed that the white pulp atrophied, the splenic corpuscles were less demolished and the lymphocytes of the spleen were reduced slightly compared with Normal.

### 3.3. Effect of TP on Biochemical Parameters in the Serum

**Figure 4:**
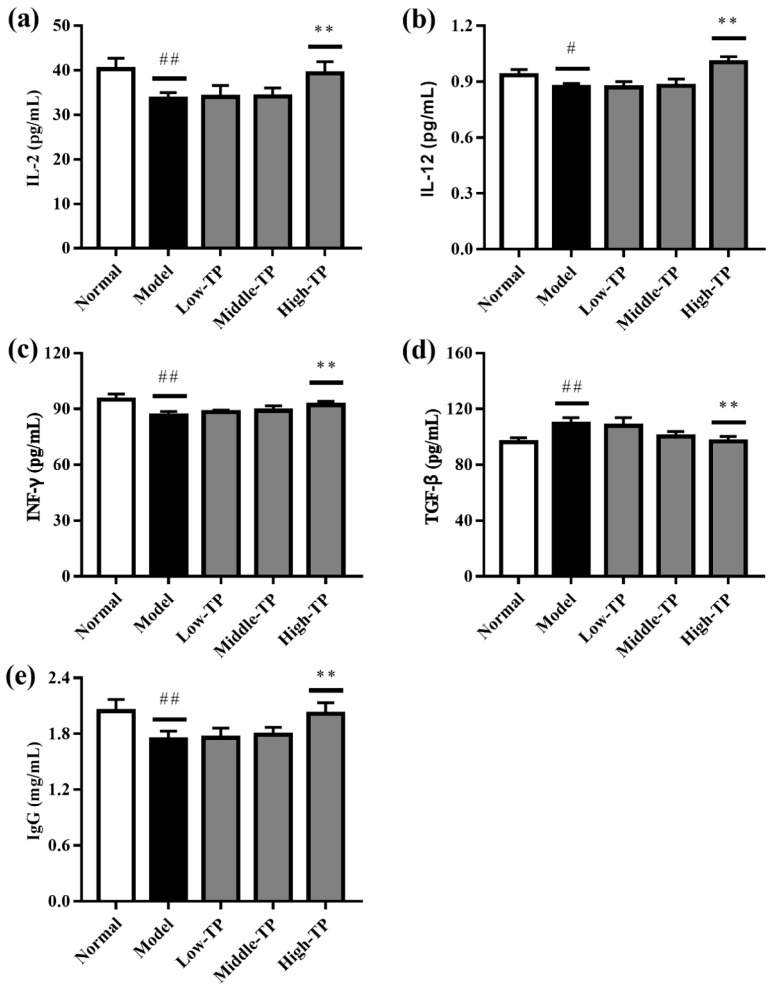
Effect of TP on IL-2 (**a**); IL-12 (**b**); INF-γ (**c**); TGF-β (**d**) and Ig G (**e**). # *p* < 0.05 versus Normal. ## *p* < 0.01 versus Normal. ** *p* < 0.01 versus Model.

As shown in Figure 4, the levels of IL-2, IL-12, INF-γ and Ig G in the Normal were significantly higher than Model (Figure 4a–c,e), and the level of TGF-β in the Normal were significantly lower than Model (Figure 4d) (# *p* < 0.05; ## *p* < 0.01). The results indicated that cyclophosphamide can significantly decrease the immunoregulatory function of mice. After TP treated, the levels of IL-2, IL-12, INF-γ, TGF-β and Ig G were increased to some extent. The effects of High-TP are better than Low-TP and Middle-TP. Compared to Model, High-TP can significantly increase the levels of IL-2, IL-12, INF-γ and Ig G, and significantly decrease the level of TGF-β (** *p* < 0.01), but Low-TP and Middle-TP cannot significantly regulate the levels of IL-2, IL-12, INF-γ, TGF-β and Ig G.

### 3.4. The Levels of Relative Gene Expression

**Figure 5:**
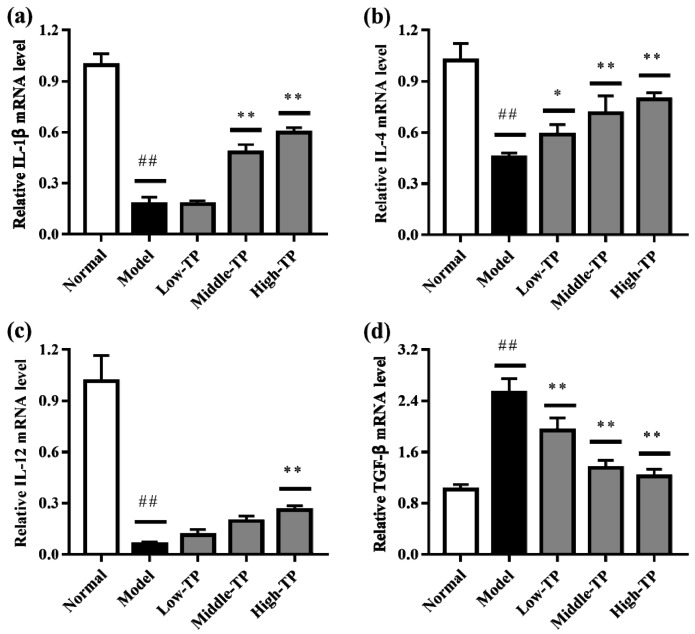
The levels of relative gene expression in liver. ## *p* < 0.01, compared to Normal. * *p* < 0.05, ** *p* < 0.01, compared to Model. (**a**) *IL-1β* expression level; (**b**) *IL-4* expression level; (**c**) *IL-12* expression level; (**d**) *TGF-β* expression level.

**Figure 6:**
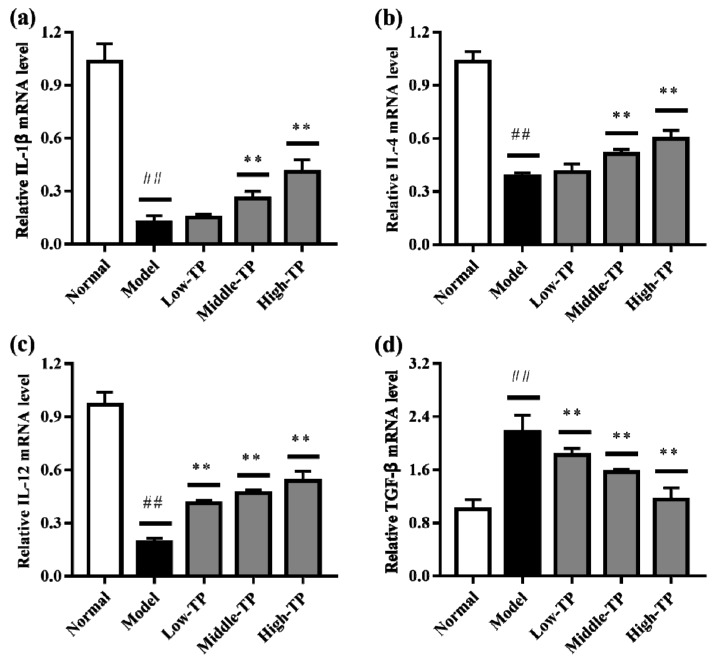
The levels of relative gene expression in spleen. ## *p* < 0.01, compared to Normal. ** *p* < 0.01, compared to Model. (**a**) *IL-1β*expression level; (**b**) *IL-4* expression level; (**c**) *IL-12* expression level; (**d**) *TGF-β* expression level.

The relative gene expression levels of *IL-1β*, *IL-4*, *IL-12* and *TGF-β* in liver (Figure 5) and spleen (Figure 6) were determined by qRT-PCR. Compared to Normal, cyclophosphamide could significantly decrease the level of IL-1β, IL-4 and IL-12 in liver and spleen (Figure 5a–c and Figure 6a–c), and significantly increase the level of TGF-β in liver and spleen (Figure 5d and Figure 6d) (## *p* < 0.01). After TP treated, the relative gene expression levels of *IL-1β*, *IL-4*, *IL-12* and *TGF-β* in liver and spleen were increased in varying degrees compared with Model. Relatively speaking, the effects of High-TP were better than Low-TP and Middle-TP. After High-TP treated, the relative gene expression levels of *IL-1β*, *IL-4* and *IL-12* in liver and spleen were increased significantly, and the relative gene expression level of *TGF-β* in liver and spleen was decreased significantly (** *p* < 0.01).

## 4. Discussion

It is well known that the remarkable feature of polysaccharides is its immune activity. TP is one of the fungus polysaccharides, which have many biological functions and no significant side effects. Furthermore, in this study, we have demonstrated that TP possessed immunity enhancing capabilities.

CY could result in immunosuppression, so mice were treated with CY to build the animal model of immunosuppression in this study. As we expected, CY significantly reduced the immune organ index and serum cytokine levels (IL-2, IL-12, INF-γ and Ig G), and improved the level of TGF-β in serum. Besides, CY markedly suppressed mRNA expression of IL-1β, IL-4 and IL-12 in liver and spleen, and promoted TGF-β expression. These results showed that the immune functions of CY-treated mice have been suppressed. In addition, this animal model could be used for evaluating the immune activity of TP.

Firstly, the reduction of immune organ index is one of the typical symptoms in mice with immunosuppression. The improvement of immune organ index indicated that the immunosuppression of mice has been mitigated. The thymus and spleen are important organs of the immune system in the body [24,25]. Compared to Model, the thymus and spleen index of TP-treated groups have been improved, and the immune activity of mice with immunosuppression have been increased.

Secondly, because of immunosuppression, the immune organs were destroyed [26,27,28,29]. Based on the photomicrographs of liver and spleen histopathology, and compared to Normal, the liver of Model showed that the arrangement of liver sinusoid and hepatic plates was disordered, massive inflammatory cells infiltrated and fatty degeneration of hepatocytes, and the spleen of Model showed that red pulp and white pulp were intermixed, splenic corpuscles demolished and disappeared, splenic sinusoid extended, and lymphocytes of spleen were reduced. After treated with TP, these features have been improved. These results indicated that TP had the functions of protecting liver and spleen, and enhancing immune defensive function.

Moreover, cytokines are synthesized and secreted by immune cells (B cells, T cells and NK cells) and non-immune cells (endothelial cells, epidermal cells and fibroblasts), which can regulate immune functions [30,31]. Among the immune cells, T cells are quite complex and heterogeneous cells, including helper T cells (Th), suppressor T cells (Ts) and cytotoxic T cells (Tc) [32]. Among them, Th cells could help enhancing the cellular and humoral immune responses, because Th cells could be differentiated into Th1 or Th2 cells by antigen stimulation, and, ultimately, Th1 or Th2 could regulate immunity response by releasing different types of cytokines [33]. For example, IL-2 could enhance the killing activity of T cells, induce T cells to secrete INF-γ, stimulate differentiation and activation of NK cells, and promote proliferation and differentiation of B lymphocytes and immune globulin production. IL-12 is a heterogenous dimer cytokines, it could promote proliferation of Th1, induce T cells and NK cells to secrete INF-γ and improve the virulent effect of NK cells. INF-γ is a high efficacy antiviral cytokine and produced by NK cells and Th1, as well as has broad spectrum immune regulative effects. TGF-β could suppress immune cells activities, differentiation of B lymphocytes, as well as INF-γ and TNF-α production [34,35,36,37]. In this study, the detection results showed that TP could up-regulated the levels of IL-2, IL-12 and INF-γ, and down-regulated the level of TGF-β, and these results indicated that TP could enhance immune activity of immunosuppressive mice. Besides, TP could up-regulate the levels of Ig G, this result also can be pointed out.

In addition, IL-1β, IL-4, TGF-β and IL-12 were closely related with immune regulation. Therefore, the mRNA levels of IL-1β, IL-4, TGF-β and IL-12 in the liver and spleen were measured. Generally speaking, IL-1β, IL-4 and IL-12 could promote immune cell functions, and can increase immunity of the body, but TGF-β could induce the production of CD4+CD25+ Treg cells, the transcription of Foxp3 and immunosuppression [38]. In this study, we demonstrated that TP can promote mRNA expression of IL-1β, IL-4 and IL-12 in liver and spleen, and suppress mRNA expression of TGF-β.

Above all, this study is good evidence to explain the protective effects of TP, but there are doubts. For instance, we are still not sure about the protection mechanism of TP, therefore, much stronger evidence is required. We should check the activity of immune cells (T-lymphocytic subgroups, macrophages cells, B cells and NK cells) after TP-treated by immunofluorescence labeling and flow cytometry technique. In addition, we should also further measure the relative proteins levels of immune regulation by western blot. Besides, the structure and character of matter decided the properties and function of matter, so finding the structure characterization and resolution of TP was also a useful way to determine its bioactive mechanism.

The immune effects of Tremella polysaccharide mainly contains two ways, one is for the non-immune system, Tremella polysaccharide could promote the gastrointestinal microbial growth and regulate the formation of intestinal microflora, meanwhile Tremella polysaccharide could enhance the immunities of exogenous pathogenic bacteria in animal; two is for the immune system, Tremella polysaccharide could enhance humoral immunity, and the phagocytic ability, Tremella polysaccharide could also improve lymphocyte activity and function, promote cell growth factor, and protect erythrocyte membrane less susceptible to oxidative damage. Thus, the immune ability of animal body is improved, and the resistance of the animal to disease is enhanced. In addition to enhancing immunity, tremella polysaccharides can also promote protein and nucleic acid synthesis, increase their ability to repair, and maintain the function of organs, especially the liver [39]. In this study, we determined a pure tremella polysaccharide which was separated from the main origin of Tremella in Sichuan, China. This tremella polysaccharide was extracted using water and purified by CTAB (cetyltrimethyl ammonium bromide) method, it was a kind of acid heteropolysaccharide. This tremella polysaccharide showed the immunomodulatory effects as the past study and the mechanism may also be similar.

## 5. Conclusions

**Figure 7:**
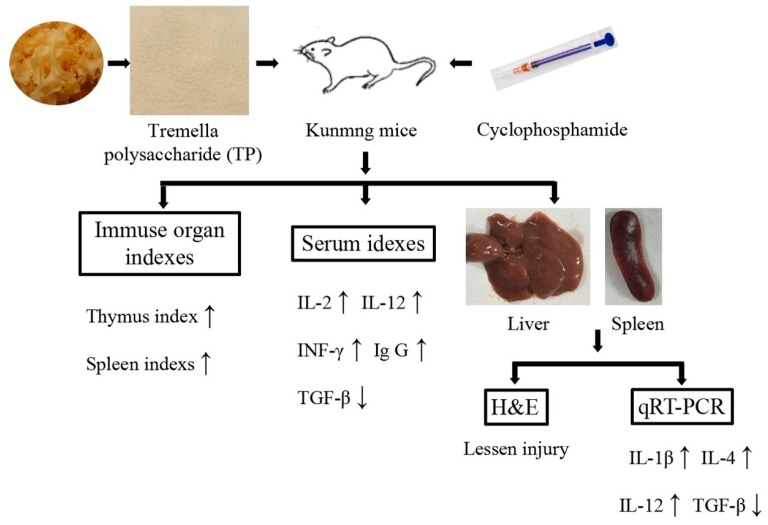
The mechanism of this study.

In conclusion, TP can protect immune organs (improving immune organ index and alleviate immune organ damage), significantly improve the levels of IL-2, IL-12, INF-γ and Ig G in serum, and reduce the level of TGF-β in serum. Furthermore, TP can also effectively regulate the expression of immune-related genes in liver and spleen (promoting *IL-1β*, *IL-4* and *IL-12* expression, and suppress *TGF-β* expression in liver and spleen). Thus, it is clear that TP possess the preventive effect for immunosuppression (Figure 7).

## Figures and Tables

**Table 1:** Primer sequences used for qRT-PCR.

Gene	Sequence (5′-3′) *	Size (bp)
*IL-1β*	GAAATGCCACCTTTTGACAGTG	116
	TGGATGCTCTCATCAGGACAG	
*IL-4*	GGTCTCAACCCCCAGCTAGT	102
	GCCGATGATCTCTCTCAAGTGAT	
*1L-12*	CTGGAGCACTCCCCATTCCTA	160
	GCAGACATTCCCGCCTTTG	
*TGF-β*	CTTCAATACGTCAGACATTCGGG	142
	GTAACGCCAGGAATTGTTGCTA	
*β-actin*	GAGAAAATCTGGCACCACACCT	172
	GCACAGCCTGGATAGCAACGTA	

* the former was forward primer; the latter was reverse primer.
